# Association of Fish Consumption with the Omega-3 Index and Vitamin D Status among Adolescent Females in the Southwest Coastal Zone of Bangladesh

**DOI:** 10.1016/j.tjnut.2026.101520

**Published:** 2026-05-06

**Authors:** Gulshan Ara, Baukje de Roos, David C Little, Abdullah-Al Mamun, Rafid Hassan, Eleanor Grieve, Samira Dilruba Ali, Tahmeed Ahmed, Nanna Roos

**Affiliations:** 1 Nutrition Research Division (NRD), International Centre for Diarrhoeal Diseases and Research, Bangladesh (icddr,b), Dhaka, Bangladesh; 2 Department of Nutrition, Exercise, and Sports, University of Copenhagen, Copenhagen, Denmark; 3 The Rowett Institute, School of Medicine, Medical Sciences and Nutrition, University of Aberdeen, Aberdeen, United Kingdom; 4 Institute of Aquaculture, University of Stirling, Stirling, United Kingdom; 5 Department of Fisheries and Marine Science, Noakhali Science and Technology University, Noakhali, Bangladesh; 6 Health Economics and Health Technology Assessment (HEHTA), Institute of Health and Wellbeing, University of Glasgow, Glasgow, United Kingdom

**Keywords:** Omega-3 fatty acids, omega-3 Index, vitamin D, adolescent female, Bangladesh

## Abstract

**Background:**

Omega-3 fatty acids (O3FA) and vitamin D are essential for growth, bone accrual, and neurodevelopment during adolescence and may affect future maternal and birth outcomes. Fish is a major dietary source of O3FA and vitamin D. However, evidence linking fish intake with O3FA and vitamin D status among adolescent females in Bangladesh remains limited.

**Objectives:**

This study aimed to investigate the associations of fish consumption with the Omega-3 Index (O3I) and serum 25-hydroxyvitamin D [25(OH)D] among adolescent females living across salinity gradients in southwest coastal Bangladesh.

**Methods:**

Two repeated cross-sectional surveys were conducted among 295 females during dry (August–September 2017) and wet (April–May 2018) seasons. Fish intake over the past week was assessed. Whole-blood eicosapentaenoic and docosahexaenoic acids were used to derive O3I, and serum 25(OH)D was measured. Associations between tertiles of fish intake and biomarker outcomes were examined using multivariable linear and logistic regression.

**Results:**

Mean O3I was 4.3% during dry season and 4.9% in wet season. Vitamin D insufficiency (<50 nmol/L) affected 25% of females during dry season and 39% in wet season. Compared with T1, adolescents in T3 of total fish intake had higher O3I (dry: *β* = 0.57; wet: *β* = 1.26) and greater odds of achieving O3I ≥4% [dry: adjusted odds ratio (AOR) = 2.12; wet: AOR = 7.98]. Tilapia consumption showed the strongest associations: females in T3 had higher O3I (dry: *β* = 1.08; wet: *β* = 1.63) and higher odds of O3I ≥4% (dry: AOR = 3.65; wet: AOR = 23.85). During wet season, higher total fish and tilapia intake were associated with higher 25(OH)D (*β* = 5.06 and 7.96) and lower odds of insufficiency for tilapia consumers (AOR = 0.25).

**Conclusions:**

Higher fish consumption, particularly tilapia, was associated with improved O3I and vitamin D status among adolescent females in coastal Bangladesh, with the strongest effects observed during wet season. Ensuring year-round access to diverse fish species, including saline-raised tilapia, may help address ω-3 and vitamin D insufficiency in this nutritionally vulnerable population.

## Introduction

Fish consumption is associated with improved growth and health because of its high-quality protein, high concentrations of long-chain omega-3 fatty acids (O3FA), and key micronutrients [1], including vitamin D [2,3]. In Bangladesh, capture fisheries and aquaculture play a central role in food security and healthy livelihoods [4,5]. The health benefits of fish are attributable, in part, to their content of long-chain O3FA, particularly DHA (22:6), and EPA (20:5). DHA is critical for brain development and cognitive function [6,7], whereas EPA has anti-inflammatory and cardioprotective properties [8,9]. Globally, inadequate intake of long-chain O3FA contributes to the burden of cardiovascular diseases (CVDs), resulting in an estimated 738,000 deaths in 2021, with mortality being particularly high in South Asia [10,11]. Regular consumption of 200–500 mg of DHA and EPA per day has been associated with reduced risk of coronary artery disease and other chronic conditions [12]. Endogenous DHA and EPA synthesis is limited in humans; therefore, adequate intake of preformed DHA and EPA is necessary [7]. Consumption of EPA and DHA directly from whole fish appears to be more effective for biological incorporation than an equivalent amount provided as supplementation [13]. The Omega-3 Index (O3I), which is the proportion of EPA and DHA in erythrocyte membranes, provides an integrated biomarker of both long-term ω-3 status and also reflects habitual fish intake [14,15].

Fish is also a major dietary source of vitamin D, and regular consumption of fish, particularly oily fish species, is associated with higher serum 25-hydroxyvitamin D [25(OH)D] concentrations [16,17]. Vitamin D plays an essential role in bone health and immune function [18], and its deficiency during pregnancy is associated with poor health outcomes of the females and newborn [19,20]. Poor vitamin D status is highly prevalent among females in Bangladesh, with reported prevalence of deficiency ranging from 38% to 100% based on serum 25(OH)D concentrations [21].

Adequate intakes of O3FA and vitamin D are particularly important for adolescent girls. Globally, ∼21 million females aged 15–19 y become pregnant each year, with a substantial proportion living in low- and middle-income countries [22]. Bangladesh has one of the highest rates of early marriage and adolescent pregnancy [23], which increases the importance of adequate nutritional reserve before and during early reproductive years. During pregnancy, maternal DHA and EPA are transferred to the fetus and later to the infant through breast milk [24]. Despite this, adolescent girls remain nutritionally vulnerable due to dietary inadequacy and gender-related inequalities.

In coastal Bangladesh, the nutrient composition of fish varies across ecological zones. A previous study documented that DHA and EPA contents in fish declined across the salinity gradient, from the oceanic high-saline coast, across the medium- and low-saline river delta to inland freshwater regions [25]. Understanding how fish consumption contributes to O3I and vitamin D status, among adolescent females across these ecological contexts, is important for informing strategies to improve intergenerational nutrition and health in coastal communities. Therefore, this study aimed to examine the association between fish consumption and both the O3I and serum 25(OH)D concentrations among adolescent females living across salinity zones in southwest coastal Bangladesh.

## Methods

### Study design and population

Two cross-sectional surveys were carried out among adolescent females aged 12–16 y in the southwest coastal floodplain of Bangladesh (Khulna, Satkhira, and Bagerhat districts). Data were collected during the dry season (August–September 2017) and again during the wet season (April–May 2018). The study design has been described in detail elsewhere [26]. Sampling was stratified across 4 agroecological salinity zones: high saline (HS), medium saline (MS), low saline (LS), and freshwater (FW). A fifth zone comprising communities around a fish-processing plant was initially included; however, because salinity conditions were comparable with those of the freshwater zone, the 2 zones were merged for analysis. Within each zone, the sample was further stratified to achieve an equal distribution of Hindu and Muslim participants, except in the fish-processing plant communities, where all participants were Muslim. An equal number of adolescent females was targeted in each salinity zone, resulting in a total sample size of 300 participants.

### Ethics approval and consent to participate

The research was carried out in accordance with the Declaration of Helsinki. Ethical approval for this study was granted by the Institutional Review Board of the International Centre for Diarrhoeal Diseases and Research, Bangladesh (icddr,b; protocol number: PR-17037) and the National Health Service (NHS), Invasive or Clinical Research (NICR) Committee, University of Stirling (NICR 16/17—paper number 82). Written informed consent was obtained from all individual participants and their parents or guardians (for study participants aged ≤16 y) before participating in this observational study.

### Data and blood sample collection

Pairs of trained female enumerators with undergraduate-level backgrounds in nutrition and food science conducted household interviews using a pretested questionnaire administered through face-to-face interviews (Supplementary File 1). Venous blood samples (5 mL) were collected by a trained phlebotomist applying standard aseptic procedures. Capillary whole blood was collected via the finger-prick method and processed as dried blood spots [26]. Participants were not required to fast before blood collection.

### Sociodemographic information

Sociodemographic information was collected at both the household and individual levels, as described elsewhere [26,27]. A household wealth index was constructed using principal component analysis, based on household assets and housing characteristics, and households were classified into quintiles: poorest, poorer, middle, richer, and richest [28].

### Dietary assessment

Dietary patterns were assessed using dietary diversity scores derived from a 24-h dietary recall [29]. Fish consumption was assessed using a previously validated 7-d dietary recall questionnaire [30], supported by a validated photo album developed for the semiquantitative fish consumption assessment in this population. The photo album contained images of cooked fish prepared according to common local recipes, and depicting different species, portion sizes, and edible parts. Participants reported their daily fish intake over the previous 7 d, including fish species, source (own production, market, or gift), and portion size. Portion sizes were reported using locally relevant units. During interviews, trained enumerators assisted participants in matching their reported intake to the most appropriate photograph to ensure correct portion size estimation. Three serving categories were defined at the household level: *1*) whole fish (mostly small fish consumed whole), *2*) filleted or pieces of large fish, and *3*) fish portions (small fish not consumed whole or as pieces). These were further subdivided into small, medium, and large size categories, with size ranges differing by species. Information was also collected on parts consumed (e.g., head and tail) and whether fish were eaten with or without soft bones; whole-body consumption was assumed for small fish. Fish weight included edible soft bones, and plate waste (e.g., head and tail remnants) was subtracted to estimate edible intake. Species-and size-specific conversion factors were then applied to convert reported intake into raw weight (g). All reported fish species were further categorized into 4 groups: crustaceans, small fish (<0.25 m in maximum length), large fish (≥0.25 m in maximum length), and tilapia [31]. Tilapia was considered a separate category because of its high consumption in the study area and its previously reported association with ω-3 status [26].

### Biomarker assessment

#### Vitamin D

Venous blood was collected in serum separator tubes and allowed to clot at room temperature. Samples were centrifuged within 2 h of collection, and serum was aliquoted into cryovials. Serum samples were stored in the field and subsequently transferred to the Nutritional Biochemistry Laboratory at icddr,b, where the serum samples were stored at −80°C until analysis. Vitamin D [total 25(OH)D] was measured by electrochemiluminescence immunoassay using the Roche automated immunoanalyzer cobas e601. The assay used a polyclonal antibody directed against 25(OH)D [27,32]. Vitamin D insufficiency was defined as serum 25(OH)D <50 nmol/L [21].

#### O3I

Dried blood spot samples were shipped to the analytical laboratory at the University of Stirling, United Kingdom, and were analyzed using gas chromatography after the standardized OmegaQuant method [33]. The O3I was expressed as the proportion of the sum of EPA and DHA to total erythrocyte fatty acids [33]. Because samples were obtained from whole blood, values were converted to erythrocyte equivalents using established correction factors [34]. The O3I was analyzed as a continuous measure and categorized into <4% (high risk), 4%–8% (intermediate risk), and >8% (low risk) based on CVD risk thresholds [15]. For regression analyses, O3I ≥4% was used as the binary outcome to indicate low CVD risk.

### Data analysis

Descriptive statistics were used to summarize participants’ characteristics across survey rounds and salinity zones. Continuous variables were presented as means (SDs) or medians (IQRs), and categorical variables as frequencies and percentages. The O3I and serum 25(OH)D concentrations were analyzed both as continuous outcomes and as binary outcomes (O3I ≥4% compared with <4%; 25(OH)D insufficiency <50 compared with ≥50 nmol/L).

Associations between fish consumption and biomarker outcomes were examined separately for the dry and wet seasons. This approach was taken because season and salinity conditions are strongly collinear in coastal Bangladesh, as seasonal changes directly affect water depth, salinity intrusion, and the availability of specific fish species. Including season as a simple covariate would, therefore, introduce instability in the models. Analyzing each season independently avoids this collinearity and allows clearer interpretation of ecological differences in fish availability and nutrient status.

Fish consumption (total and species-specific) was categorized into tertiles (T1 = lowest, T3 = highest) based on grams consumed in the previous 7 d. For each season, 4 models were estimated for the O3I:

1. Model I: linear regression of total fish intake on O3I;
2. Model II: linear regression of species-specific fish intake on O3I;
3. Model III: logistic regression of total fish intake on O3I ≥4%; and
4. Model IV: logistic regression of species-specific fish intake on O3I ≥4%.

The same framework (Models I–IV) was applied to serum 25(OH)D (continuous and insufficiency <50 nmol/L) as outcomes. All models were adjusted for age, religion, dietary diversity score, salinity zone, and household wealth index following previous literature [26]. Linear regression model’s results were reported as regression coefficients (*β*) with 95% confidence intervals (CIs), and logistic regression results were reported as adjusted odds ratios (AOR) with 95% CIs.

Model performance and assumptions were checked systematically. Multicollinearity was evaluated using variance inflation factors <10. For linear regression, the coefficient of determination (*R*^2^) was assessed alongside heteroskedasticity using the Breusch–Pagan or Cook–Weisberg test, and where present, robust SEs were applied. Residuals were inspected visually using kernel density plots, normal probability plots, and the Shapiro–Wilk test. Model influence diagnostics were conducted using Cook’s distance (<1). For logistic regression models, overall model fit was assessed using the Hosmer–Lemeshow goodness-of-fit test, and model performance was evaluated using pseudo-*R*^*2*^ statistics. Statistical significance was defined as a *P* value < 0.05. All analyses were performed using Stata version 15.0 (StataCorp).

## Results

### Sociodemographic characteristics and fish intake patterns

We excluded the participants with missing fish intake and biomarker data, and the final analytic sample included 295 adolescent females in the dry season and 260 in the wet season. The mean age was 13.9 y, and most females had completed secondary-level education. Dietary diversity score was higher in the wet season (*P* < 0.001). The median intake of tilapia was highest in the MS zone during both seasons (*P* < 0.001), whereas crustacean intake was largest in the HS zone (*P* < 0.001). Small fish intake was higher in the HS zone during the dry season (*P* = 0.002) and in the MS zone during the wet season (*P* < 0.001). On the other hand, the intake of large fish peaked in the LS zone in both seasons (*P* < 0.001) (Table 1).

**TABLE 1:** Background characteristics and fish consumption of adolescent females (12–16 y) by seasons and salinity zones

Characteristics	Overall (*n* = 295)^3^	*P* value^1^	Salinity zones				*P* value^2^
			HS (*n* = 60)^3^	MS (*n* = 60)^3^	LS (*n* = 55)^3^	FW (*n* = 120)^3^	
Age (y)	13.9 ± 1.4		14.0 ± 1.5	13.9 ± 1.4	14.2 ± 1.5	13.7 ± 1.5	0.209
Education level							
≤Primary	54 (18.3)		10 (16.7)	11 (18.3)	11 (20.0)	22 (18.3)	0.975
Secondary	241 (81.7)		50 (83.3)	49 (81.7)	44 (80.0)	98 (81.7)	
Religion							
Muslim	175 (59.3)		28 (46.7)	31 (51.7)	26 (47.3)	90 (75)	<0.001
Hindu	120 (40.7)		32 (53.3)	29 (48.3)	29 (52.7)	30 (25)	
Wealth index							
Poorest	59 (20.0)		10 (16.7)	23 (38.3)	12 (21.8)	14(11.7)	0.001
Poorer	59 (20.0)		11 (18.3)	8 (13.3)	12 (21.8)	28 (23.3)	
Middle	61 (20.7)		12 (20.0)	11 (18.3)	13 (23.6)	25 (20.8)	
Richer	57 (19.3)		19 (31.7)	12 (20.0)	6 (10.9)	20 (16.7)	
Richest	59 (20.0)		8 (13.3)	6 (10.0)	12 (21.8)	33 (27.5)	
Dietary diversity score							
Dry (*n =* 295)	3.8 (0.9)	<0.001	3.9 (0.8)	3.7 (0.8)	4.3 (1.0)	3.6 (0.8)	0.001
Wet (*n =* 260)	5.1 (1.2)		5.1 (1.1)	5.1 (1.2)	5.3 (1.1)	5.1 (1.3)	0.581
Tilapia intake (g/wk)							
Dry (*n =* 295)	260 (145, 425)	0.198	290 (200, 459.5)	330 (227.5, 585)	300 (150, 431)	196 (120, 330)	<0.001
Wet (*n =* 260)	272.5 (75, 476)		240 (105, 490)	462 (315, 627.5)	115 (0, 320)	233 (47.5, 437.5)	<0.001
Crustacean intake (g/wk)							
Dry (*n =* 295)	64.4 ± 135.5	0.057	92.5 ± 183.0	65 (0, 205)	59.5 ± 178.1	27.3 ± 49.4	<0.001
Wet (*n =* 260)	63.4 ± 107.2		72.5 ± 116	65 (0, 162.5)	47.8 ± 81.5	42.6 ± 92.5	<0.001
Large fish intake (g/wk)							
Dry (*n =* 295)	60 (0, 145)	0.285	57.2 ± 78.8	35.8 ± 72.1	130 (70, 255)	80 (0, 162.5)	<0.001
Wet (*n =* 260)	55 (0, 122.5)		70 (10, 110)	34.2 ± 65.4	80 (0, 195)	60 (0, 142.5)	<0.001
Small fish intake (g/wk)							
Dry (*n =* 295)	20 (0, 85)	<0.001	54.8 ± 98.5	22.5 (0, 75)	28.7 ± 50.5	40 (0, 110)	0.002
Wet (*n =* 260)	38.5 ± 107.4		12.6 ± 23.7	30.7 ± 62.7	9.8 ± 32.5	20 (0, 75)	<0.001
Total fish intake (g/wk)							
Dry (*n =* 295)	470 (310, 677)	0.048	477 (292.5, 677)	580.5 (377.5, 797.5)	570 (295, 720)	403.5 (290, 617.5)	0.002
Wet (*n =* 260)	445 (245, 660)		440 (260, 619)	642.5 (432.5, 857.5)	225 (145, 570)	415 (260, 634.5)	<0.001

Abbreviations: FW, freshwater; HS, high saline; LS, low saline; MS, medium saline.

Data presented as mean ± SD, or median (IQR), where the IQR represents the 25th and 75th percentiles, and as *n* (%) where applicable. Mean ± SD was also used in cases where median values were zero.

1 *P* values were derived using the Wilcoxon rank-sum test for continuous variables to compare differences between the 2 seasons.

2 *P* values were derived using the Kruskal–Wallis test for continuous variables and the χ^2^ test of independence for categorical variables to compare differences among the 4 salinity zones.

3 Number of participants during baseline (dry season).

### ω-3 Fatty acid and 25(OH)D status of adolescent females

Adolescent females showed clear seasonal and salinity-related differences in ω-3 and vitamin D status. The median O3I was significantly higher during the wet compared with the dry season (4.85% compared with 4.35%, *P* < 0.001). Across salinity zones, O3I differed significantly (*P* < 0.001), with the highest median values observed in the MS zone in both seasons (dry: 5.15%; wet: 6.24%). The lowest O3I was observed in the FW zone during the dry season (3.79%) and in the LS zone in the wet season (3.79%).

In contrast, median serum 25(OH)D concentrations were significantly lower in the wet season compared with the dry season (55.6 compared with 60.7 nmol/L, *P* < 0.001). The prevalence of 25(OH)D insufficiency increased from 25.4% in the dry season to 38.5% in the wet season (*P* = 0.001). A significant difference was observed across salinity zones (*P* < 0.001), with the lowest 25(OH)D concentrations and the highest prevalence of insufficiency in FW areas in both seasons (dry: 39.2%; wet: 57.7%) (Table 2).

**TABLE 2:** ω-3 Fatty acid and vitamin D status of adolescent females (12–16 y) across seasons and salinity zones

Fatty acid composition	Overall	*P* value^1^	Salinity zones				
			HS	MS	LS	FW	*P* value^2^
EPA (%)							
Dry (*n =* 295)	0.45 (0.31, 0.61)	0.787	0.46 (0.34, 0.68)	0.62 (0.48, 0.83)	0.36 (0.28, 0.53)	0.38 (0.28, 0.51)	<0.001
Wet (*n =* 260)	0.45 (0.31, 0.65)		0.41 (0.31, 0.53)	0.62 (0.43, 0.82)	0.31 (0.26, 0.52)	0.44 (0.31, 0.73)	<0.001
DHA (%)							
Dry (*n =* 295)	2.76 (2.24, 3.17)	<0.001	2.64 (2.36, 2.99)	3.25 (2.85, 3.55)	2.86 (2.29, 3.14)	2.45 (1.98, 2.92)	<0.001
Wet (*n =* 260)	3.19 (2.72, 3.69)		2.96 (2.62, 3.38)	3.94 (3.53, 4.31)	2.50 (1.89, 2.98)	3.22 (2.85, 3.54)	<0.001
EPA + DHA in whole blood (%)							
Dry (*n =* 295)	3.25 (2.65, 3.76)	<0.001	3.24 (2.67, 3.64)	3.83 (3.39, 4.47)	3.25 (2.74, 3.71)	2.85 (2.36, 3.38)	<0.001
Wet (*n =* 260)	3.61 (3.03, 4.36)		3.38 (2.94, 3.86)	4.61 (4.06, 5.06)	2.85 (2.20, 3.45)	3.64 (3.18, 4.25)	<0.001
O3I (%)							
Dry (*n =* 295)	4.35 (3.51, 5.06)	<0.001	4.33 (3.54, 4.89)	5.15 (4.54, 6.04)	4.35 (3.64, 4.99)	3.79 (3.11, 4.52)	<0.001
Wet (*n =* 260)	4.85 (4.04, 5.89)		4.53 (3.92, 5.19)	6.24 (5.47, 6.86)	3.79 (2.89, 4.63)	4.89 (4.25, 5.73)	<0.001
25(OH)D concentration (nmol/L)							
Dry (*n =* 295)	60.7 (49.4, 70.8)	<0.001	60.7 (55.7, 71.6)	73.6 (62.8, 87.8)	62.0 (49.7, 70.5)	53.6 (43.6, 62.6)	<0.001
Wet (*n =* 260)	55.6 (44.0, 65.9)		59.1 (49.2, 69.1)	70.0 (59.6, 83.9)	54.6 (43.0, 65.0)	46.3 (38.0, 57.1)	<0.001
25(OH)D insufficiency							
Dry (*n =* 295)	75 (25.4)	0.001	10 (16.7)	4 (6.7)	14 (25.5)	47 (39.2)	<0.001
Wet (*n =* 260)	100 (38.5)		14 (25.5)	7 (13.5)	19 (38.8)	60 (57.7)	<0.001

Abbreviations: FW, freshwater; HS, high saline; LS, low saline; MS, medium saline; O3I, Omega-3 index (EPA + DHA in erythrocytes); 25(OH)D, 25-hydroxyvitamin D.

Data presented as median (IQR), and as *n* (%) where applicable.

EPA and DHA were measured from dried blood spots. EPA + DHA in whole blood represents the sum of the EPA and DHA in whole blood.

Serum 25 (OH)D was used to assess vitamin D status. Vitamin D insufficiency was considered as serum 25 (OH)D <50 nmol/L.

1 *P* values were derived using the Wilcoxon rank-sum test for continuous variables and the χ^2^ test of independence for categorical variables to compare differences between the 2 seasons.

2 *P* values were derived using the Kruskal–Wallis test for continuous variables and the χ^2^ test of independence for categorical variables to compare differences among the 4 salinity zones within each season.

**FIGURE 1:**
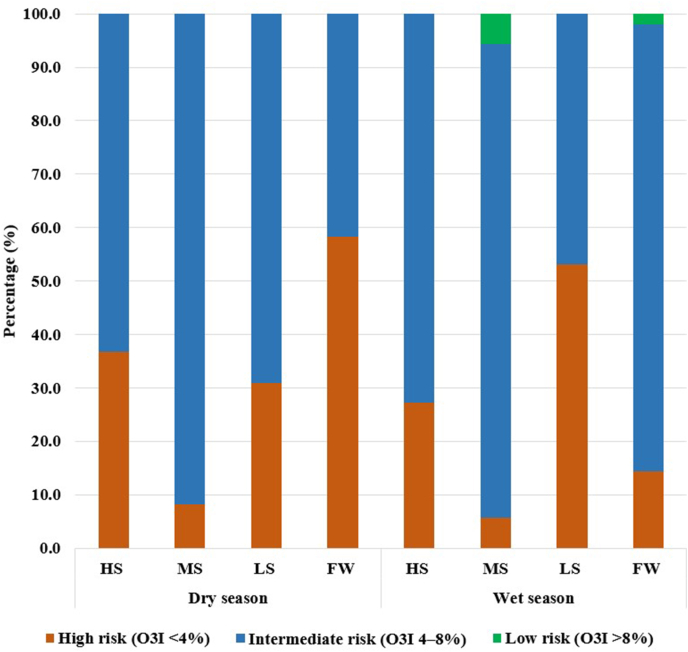
Distribution of O3I categories by cardiovascular disease risk across salinity zones and seasons. FW: Freshwater, HS: High saline, LS: Low saline, MS: Medium saline, O3I: Omega-3 Index.

Figure 1 shows the distribution of the O3I categories across salinity zones and seasons. Most females were in the intermediate range (4%–8%), particularly in the MS zone (92% in the dry and 89% in the wet season). A low O3I (<4%) was most common in the FW (58% in dry) and LS zones (53% in wet). Only a small proportion of females in the MS (5.8%) and FW (1.9%) zones had an O3I >8% during the wet season.

### Association between fish consumption and O3I

The relationship between fish intake and O3I is illustrated as scatterplots for both the dry and wet seasons **(**Supplementary File 2, Supplementary Figures 1–5**)**. Furthermore, Table 3 presents the adjusted associations between weekly fish consumption and the O3I in dry and wet seasons. It was observed that the total fish intake was positively associated with O3I in both seasons. Females in the highest tertile of total fish intake (T3) had a higher O3I compared with those in the lowest tertile (T1) (*β* = 0.57, 95% CI: 0.30, 0.84, *P* < 0.001) during the dry season. The adolescent females also had higher odds of achieving an O3I ≥4% (AOR = 2.12, 95% CI: 1.09, 4.11, *P* = 0.027). These associations were even stronger in the wet season (*β* = 1.26, 95% CI: 0.94, 1.59, *P* < 0.001; AOR = 7.98, 95% CI: 2.69, 23.66, *P* < 0.001).

**TABLE 3:** Association between weekly fish consumption and O3I among adolescent females (12–16 y) across seasons

Characteristics	O3I		O3I ≥4%	
	Model I	Model II	Model III	Model IV
	Coef. (95% CI)	Coef. (95% CI)	AOR (95% CI)	AOR (95% CI)
Dry season (*n =* 295)				
Total fish intake				
T1 (≤347 g)	Ref.		Ref.	
T2 (348–615 g)	0.27 (−0.01, 0.55)		1.61 (0.86, 3.03)	
T3 (≥616 g)	0.57 (0.30, 0.84)∗∗∗		2.12 (1.09, 4.11)∗	
Tilapia intake				
T1 (≤180 g)		Ref.		Ref.
T2 (181–357 g)		0.68 (0.42, 0.95)∗∗∗		3.12 (1.57, 6.18)∗∗
T3 (≥358 g)		1.08 (0.81, 1.35)∗∗∗		3.65 (1.82, 7.32)∗∗∗
*R*^2^ (%)	29.60	40.80	16.50	20.40
Wet season (*n =* 260)				
Total fish intake				
T1 (≤300 g)	Ref.		Ref.	
T2 (301–590 g)	0.86 (0.54, 1.17)∗∗∗		2.67 (1.18, 6.07)∗	
T3 (≥590 g)	1.26 (0.94, 1.59)∗∗∗		7.98 (2.69, 23.66)∗∗∗	
Tilapia intake				
T1 (≤135 g)		Ref.		Ref.
T2 (136–378 g)		1.14 (0.83, 1.45)∗∗∗		7.53 (2.82, 20.13)∗∗∗
T3 (≥379 g)		1.63 (1.31, 1.94)∗∗∗		23.85 (6.25, 90.95)∗∗∗
*R*^2^ (%)	49.30	56.50	31.70	40.26

Abbreviations: AOR, adjusted odds ratio; CI, confidence interval; Coef, coefficient; O3I, Omega-3 Index; Ref, reference; T, tertiles of fish consumption.

Models I–II: linear regression (continuous O3I); Models III–IV: logistic regression (O3I ≥ 4%). All models also adjusted for age, religion, dietary diversity, salinity zone, and wealth index. *R*^2^ indicates variance explained. ∗*P* < 0.05; ∗∗*P* < 0.01; ∗∗∗*P* < 0.001.

Tilapia intake showed similar and stronger associations. Females in the highest tilapia intake tertile (T3) had higher O3I (dry: *β* = 1.08, 95% CI: 0.81, 1.35, *P* < 0.001; wet: *β* = 1.63, 95% CI: 1.31, 1.94, *P* < 0.001) and greater odds of having an O3I ≥4% (dry: AOR = 3.65, 95% CI: 1.82, 7.32, *P* < 0.001; wet: AOR = 23.85, 95% CI: 6.25, 90.95, *P* < 0.001) compared with T1. In the dry season, moderate consumption of small fish (T2) was also positively associated with O3I (*β* = 0.36, 95% CI: 0.09, 0.63, *P* = 0.009), whereas no significant association was observed for crustaceans or large fish (Supplementary File 2, Supplementary Table 1).

### Association between fish consumption and serum 25(OH)D status

Table 4 shows the associations between weekly fish intake and vitamin D status. Only in the wet season, higher total fish intake was significantly associated with improved vitamin D status: females in T3 of total fish intake had higher serum 25(OH)D concentrations (*β* = 5.06, 95% CI: 0.54, 9.59, *P* = 0.028) and lower odds of 25(OH)D insufficiency (AOR= 0.48, 95% CI: 0.23, 1.00, *P* = 0.050) compared with T1.

**TABLE 4:** Association between weekly fish consumption and vitamin D status among adolescent females (12–16 y) across seasons

Characteristics	Serum 25(OH)D concentration (nmol/L)		25(OH)D insufficiency (<50 nmol/L)	
	Model I	Model II	Model III	Model IV
	Coef. (95% CI)	Coef. (95% CI)	AOR (95% CI)	AOR (95% CI)
Dry season (*n =* 295)				
Total fish intake				
T1 (≤347 g)	Ref.		Ref.	
T2 (348–615 g)	−3.74 (−7.81, 0.33)		1.22 (0.63, 2.37)	
T3 (≥616 g)	−0.21 (−4.77, 4.35)		0.79 (0.39, 1.63)	
Tilapia intake				
T1 (≤180 g)		Ref.		Ref.
T2 (181–357 g)		6.92 (2.78, 11.06)∗∗		0.48 (0.23, 1.03)
T3 (≥358 g)		0.93 (−3.7, 5.57)		1.05 (0.52, 2.11)
*R*^*2*^ (%)	26.40	30.00	10.37	12.86
Wet season (*n =* 260)				
Total fish intake				
T1 (≤300 g)	Ref.		Ref.	
T2 (301–590 g)	4.38 (−0.05, 8.81)		0.68 (0.34, 1.36)	
T3 (≥590 g)	5.06 (0.54, 9.59)∗		0.48 (0.23, 1.00)	
Tilapia intake				
T1 (≤135 g)		Ref.		Ref.
T2 (136–378 g)		4.50 (0.29, 8.72)∗		0.66 (0.31, 1.42)
T3 (≥379 g)		7.96 (3.49, 12.42)∗∗		0.25 (0.11, 0.57)∗∗
*R*^2^ (%)	35.80	39.70	17.05	22.25

Abbreviations: AOR, Adjusted odds ratio; CI, Confidence interval; Coef, Coefficient; Ref, Reference; T, Tertiles of fish consumption; 25(OH)D, 25-hydroxyvitamin D.

Models I–II: linear regression (continuous serum vitamin D concentration); Models III–IV: logistic regression (vitamin D insufficiency). All models also adjusted for age, religion, dietary diversity, salinity zone, and wealth index. *R*^2^ indicates variance explained. ∗*P* < 0.05; ∗∗*P* < 0.01; ∗∗∗*P* < 0.001.

Consumption of tilapia showed the strongest association with 25(OH)D. Moderate tilapia intake (T2) during the dry season was associated with higher serum 25(OH)D concentrations (*β* = 6.92, 95% CI: 2.78, 11.06, *P* = 0.001), although the odds of insufficiency were not significantly reduced (AOR = 0.48, 95% CI: 0.23, 1.03, *P* = 0.058). In the wet season, higher tilapia intake (T3) was associated with higher 25(OH)D concentrations (*β* = 7.96, 95% CI: 3.49, 12.42, *P* = 0.001) and lower odds of 25(OH)D insufficiency (AOR = 0.25, 95% CI: 0.11, 0.57, *P* = 0.001). Moderate consumption of small fish (T2) in the wet season was also associated with lower odds of 25(OH)D insufficiency (AOR= 0.21, 95% CI: 0.06, 0.78, *P* = 0.020), whereas no significant associations were observed for crustaceans or large fish (Supplementary File 2, Supplementary Table 2).

## Discussion

We found that adolescent females living in the southwest coastal zones of Bangladesh had seasonal and geographic variations in fish consumption. Fish intake was frequently reported across all areas, with the highest consumption observed in MS zones (dry season: 580 g/wk; wet season: 643 g/wk). Both the O3I and 25(OH)D concentrations also showed variations across seasons and salinity zones. Most females (70%) had an intermediate risk O3I (4%–8%), whereas 25(OH)D insufficiency ranged from 25% in the dry season to 39% in the wet season. Higher total fish intake, especially tilapia, in both seasons was significantly associated with an elevated O3I. Although vitamin D concentrations varied across ecological zones and seasons, the pattern of association between fish intake and 25(OH)D was less consistent.

The O3I of these adolescents ranged from 4.3% to 4.9% in the dry and wet seasons, respectively, which falls within the intermediate risk range for CVDs. These values are higher than those reported for neighboring country India (3.62%), but comparable to the 4%‒6% range observed in most countries [35]. Differences in fish consumption patterns likely explain this cross-country variation. For instance, the mean daily per capita fish intake in India is ∼17 g, ∼4 times lower than that in Bangladesh [36,37]. This aligns with global evidence linking dietary EPA and DHA intake from seafood to ω-3 status, where populations with lower seafood consumption (e.g., India, Brazil, and the Middle East) tend to have lower O3I, whereas those with higher seafood intake (e.g., Norway, Finland, South Korea, and Japan) achieve optimal levels [35,38,39]. Within Bangladesh, our findings are consistent with an earlier study in the same coastal population, likely reflecting the continued availability and regular consumption of ω-3–rich foods in this region [30].

Fish consumption, especially tilapia consumption, was significantly associated with the O3I, and this effect was more evident during the wet season. Although overall fish consumption was comparable between seasons, greater tilapia intake during the wet season may help to explain the stronger association. Indeed, tilapia dominated the diet in these coastal communities, contributing roughly 60% of total fish intake, consistent with previous studies [40]. Importantly, as shown in Table 2, much of the increase in the O31 was driven by higher DHA rather than EPA, suggesting that even modest concentrations of DHA contained in fish such as tilapia can meaningfully influence whole-blood DHA status when consumed frequently and in substantial quantities [41]. For example, adolescent females in the MS zone, who consumed most tilapia, had the highest O3I, indicating a potential dose–response relationship between tilapia intake and blood ω-3 concentrations. Access patterns further reinforced these differences: most households in higher salinity areas farm tilapia as a byproduct of export-oriented aquaculture and consume it directly from their own production, whereas households in lower salinity areas rely on market purchases, limiting availability and contributing to lower tilapia intake and consequently lower O3I [30].

Globally, tilapia has become a dominant aquaculture species, although concerns have been raised about its relatively low content of EPA and DHA compared with traditional oily fish [42]. Nevertheless, the fatty acid composition of tilapia can vary depending on environment, feed, and seasons [43]. An earlier study in Bangladesh found that high saline-raised tilapia contained substantially higher amounts of EPA and DHA than freshwater-raised tilapia [30]. Thus, even though tilapia is a lean fish, the combination of large quantities consumed, higher nutrient density in saline environments, and greater accessibility makes it a meaningful contributor to ω-3 intake, particularly during the wet season in medium and high salinity areas. Another point to be noted is that the O3I reflects the fatty acid composition over the preceding 3 mo [14]. Therefore, wet season O3I values likely capture the cumulative effects of relatively higher fish intake during the late dry season, together with increased tilapia consumption in the wet season. Small fish also contributed to ω-3 intake, though their effects were weaker and less consistent across seasons. Nonetheless, the recognized role of small fish as key dietary sources of micronutrients in freshwater systems reinforces the nutritional value of maintaining dietary diversity alongside tilapia [44,45].

Consistent with evidence from meta-analyses, serum 25(OH)D concentrations were influenced by fish intake in this study [46]. Fish provide higher amounts of 25(OH)D than other dietary sources, such as egg yolk, dairy products, and mushrooms [16,47]. Although 25(OH)D content varies across species, both fatty and lean fish contribute substantially to dietary sources of 25(OH)D, as fish can store 25(OH)D in the liver and muscle fat tissues [16,17,47]. Seasonal effects were evident in this study: during the wet season, limited sunlight exposure increased reliance on dietary sources, thus fish intake appeared particularly important during this season [48]. Tilapia, which contains relatively high 25(OH)D compared with many freshwater species [17], also significantly contributed to 25(OH)D concentrations in this season. Conversely, lower tilapia consumption in low-salinity areas was associated with higher rates of insufficiency, echoing findings from an earlier study [27]. Small fish, which are well-recognized for their micronutrient contribution [44], also appeared to improve 25(OH)D status in this study, particularly in the wet season.

This is the first study to assess the relationship between fish consumption and both the O3I and 25(OH)D status among adolescent females in the coastal region of Bangladesh. A major strength of the study is its design, which captured the impact of both seasonality and salinity by following the same cohort. In addition, the quantitative assessment of fish intake using a validated semiquantitative food frequency questionnaire provides reliable estimates of actual consumption. However, this study has several limitations. There was some loss of participants during the second round of data collection, which reduced the sample size. However, sensitivity analyses indicated that this attrition did not meaningfully influence the interpretation of the results (Supplementary File 2, Supplementary Tables 3–5). The study was also conducted in a unique aquaculture-dominated region, which limits generalizability to noncoastal areas. Moreover, the nutrient content of fish, such as tilapia, can differ between high-salinity coastal ponds and freshwater systems in other regions; thus, there is a need for similar studies in noncoastal settings. Furthermore, some relevant factors, including other dietary sources of O3FA, genetic variation affecting fatty acid metabolism, and lifestyle-related factors, were not considered. These factors may have influenced the observed associations, and residual confounding cannot be ruled out. Finally, the findings must be interpreted in the broader structural context of these communities, where export-oriented shrimp and prawn farming dominate local livelihoods, and potentially influence the availability of fish for household consumption.

In conclusion, this study indicates that fish consumption plays a critical role in the O3I and 25(OH)D status among adolescent females in coastal Bangladesh, especially during the wet season. Seasonal and geographic variations influenced the intake of fish, with medium- and high-salinity areas showing higher consumption reflected in the status. Tilapia, the most frequently consumed species, showed the strongest association with O3I and 25(OH)D concentrations. Tilapias are essentially an accessible and affordable coproduct of a low-input farming system in which the target species is black tiger shrimp, a luxury mainly exported product [49]. Promoting regular tilapia intake through sustainable aquaculture and enhancing access to locally available fish could potentially help address nutrient deficiencies.

## Author contributions

The authors’ responsibilities were as follows – GA, BdR, DCL, A-AM, EG, TA, NR: designed research; GA, BdR, DCL, A-AM, EG, NR: conducted research; GA, RH, SDA: analyzed data; GA, RH, NR, BdR, A-AM, DCL: wrote the paper; GA: had primary responsibility for the final content; and all authors: read and approved the final manuscript.

## Data availability

Data described in the manuscript, code book, and analytic code will be made available upon request to the corresponding author, GA (gulshan.ara@icddrb.org). Data access is governed by the icddr,b Data Access Policy and requires approval of a Data Licensing Application and Agreement by the icddr,b Data Repository Committee (DRC) (see policy details at https://www.icddrb.org/storage/file/Document/66d3e78270ffe.pdf). Researchers who meet the criteria for access to confidential data may apply to the DRC for access.

## Declaration of generative AI and AI-assisted technologies in the writing process

During the preparation of this work, the authors used ChatGPT and Grammarly to improve readability, language, and grammar correction. After using these tools, the authors reviewed and edited the content as needed and take full responsibility for the content of the publication.

## Funding

This work is funded through the Innovative Methods and Metrics for Agriculture and Nutrition Action (IMMANA) program, led by theLondon School of Hygiene and Tropical Medicine (LSHTM). IMMANA is cofunded with UK Aid from the UK government and by the Bill & Melinda Gates Foundation INV-002962 / OPP1211308. Under the grant conditions of the Foundation, a Creative Commons Attribution 4.0 Generic License has already been assigned to the Author Accepted Manuscript version that might arise from this submission. Also, this survey was funded by AQUAFOOD, Danida, Ministry of Foreign Affairs, Denmark (grant file number 23-15-KU).

## Conflict of interest

The authors report no conflict of interests.
