# Supplementary material for: Association of Fish Consumption with the Omega-3 Index and Vitamin D Status among Adolescent Females in the Southwest Coastal Zone of Bangladesh
Source: J Nutr. 2026 May 6;156(6):101520. doi: 10.1016/j.tjnut.2026.101520 (PMC13279382; doi:10.1016/j.tjnut.2026.101520)
Supplement: Multimedia component 1 [file mmc1.docx]

| **Association of fish consumption and nutritional status among adolescent girls living in different agro-ecological zones in South West Bangladesh** |
| --- |

Consent Form (English)

**Protocol title: Association of fish consumption and nutritional status among adolescent girls living in different aquatic ecological zones in South Western Bangladesh**

Name of PI: Dr. Abdullah-Al Mamun, (NSTU) and Mrs Gulshan Ara (ICDDR,B)

We are here from the icddr’b and Noakhali Science and Technology University (NSTU) working under the lead of University of Stirling, UK in a multinational research project to collect data on food and nutritional security of adolescent girls at the household level of the south-western coastal belt of Bangladesh. We will transform your personal information into the code and keep it confidential and destroy after finishing the research. During the blood sample collection of adolescent girls and nutritional outcomes study female health assistant/experienced women will deploy. It will take at least 3-4 hours, and your information should be accurate and if you wish you can withdraw your participation from this volunteer survey work. Your participation and information may help the policy makers to formulate strategies which eventually provide benefit to the mass people.

Interviewer of this research work has clearly explained the aim and objectives of this survey, and I can withdraw my participation at any moment if I wish. I have the right to know anything relevant to this survey work.

| Does the interviewee agree? |  | Doesn’t agree |  |
| --- | --- | --- | --- |
| Name of the interviewee: |  | Signature: |  |

The interviewee came to know about the aim and objectives of this survey and willingly participate and giving his/her consent by signing in respective space.

| Name of the Interviewer: |  | Signature |  |
| --- | --- | --- | --- |

| Gulshan Ara  Assistant Scientist  Nutrition and Clinical Services Division (NCSD)  icddr,b  Mohakhali, Dhaka-1212, Bangladesh  Tel: 880-2- 8860523-32, 019259-02383/01534-328559  E-mail: gulshan.ara@icddrb.org  Website: [http://www.icddrb.org](http://www.icddrb.org/) | **Abdullah-Al Mamun PhD**  Department of Fisheries and Marine Science  Noakhali Science and Technology University  Sonapur, Noakhali-3814, Bangladesh  Cell: +8801712928710,  E-mail: mamun_au22@yahoo.com |
| --- | --- |

ICDDR,B Ethical Review Committee, **Mr. M.A. Salam Khan**, Committee Coordination

Secretariat, ICDDR,B, Mohakhali, Dhaka-1212, Bangladesh, Phone: 9886498 (direct) or 01711428989 (mobile).

**INTERVIEWER ID: /__/__/ STUDY ID: /__/__/__/**

| **HOUSEHOLD QUESTIONNAIRE FORM** | | | | |  |
| --- | --- | --- | --- | --- | --- |
| **IDENTIFICATION** | | | | |  |
| NAME OF HOUSEHOLD HEAD: | | | | |  |
| NAME OF RESPONDENT: | | | | |  |
| ADOLESCENT NAME : ADDOLESCENT LINE NO: | | | | |  |
| VILLAGE: UNION: | | | | |  |
| THANA: DISTRICT: | | | | |  |
| AREA: 01 = HS, 02 = MS, 03 = LS, 04 = FW, 05 =VC WELL BEING : 01 = BO, 02 = WO | | | | |  |
| DETAIL ADDRESS:  PHONE NO: | | | | |  |
| INTERVIEWER’S VISITS | | | | | |
| VISIT NO | VISIT-1 | VISIT-2 | VISIT-3 | FINALVISIT AND RESUILT | |
| DATE |  |  |  |  | |
| INTERVIEWER NAME |  |  |  |  | |
| SUPERVISOR NAME |  |  |  |  | |
| RESUILT |  |  |  |  | |

RESUILT CODES:

1. COMPLETED 2. NOT AT HOME 3. POSTPONED

4. REFUSED 5. PARTLY COMPLTED 6. RESPONDENT INCAPACITATED

7. OTHERS ------------------------

(SPECIFY, IF ANY PROBLEM ENCOUTERED)

| 1. **Socio Demographic Information: Household level**   Now I will ask you some questions about you and your family mebers | | | | | | | | | | | | |
| --- | --- | --- | --- | --- | --- | --- | --- | --- | --- | --- | --- | --- |
| **1.** | | Please enlist the name of the all household members (Start from the household head) | What is the relationship of (Name) to the head of the household? | | (Name) Male or female | (Name) Stay in household at night | (Name) generally eat in this household | | (Name) Age? If age <1 then write (00) | | Education level (in year) | Occupation |
| **Sl.No** | | a | b | | c | d | e | | f | | g | h |
| **1** | |  |  | | Female=01  Male=02 | 01 =Yes  02 = No | 01 =Yes  02 = No | |  | |  |  |
| **2** | |  |  | | Female=01  Male=02 | 01 =Yes  02 = No | 01 =Yes  02 = No | |  | |  |  |
| **3** | |  |  | | Female=01  Male=02 | 01 =Yes  02 = No | 01 =Yes  02 = No | |  | |  |  |
| **4** | |  |  | | Female=01  Male=02 | 01 =Yes  02 = No | 01 =Yes  02 = No | |  | |  |  |
| **5** | |  |  | | Female=01  Male=02 | 01 =Yes  02 = No | 01 =Yes  02 = No | |  | |  |  |
| **6** | |  |  | | Female=01  Male=02 | 01 =Yes  02 = No | 01 =Yes  02 = No | |  | |  |  |
| **7** | |  |  | | Female=01  Male=02 | 01 =Yes  02 = No | 01 =Yes  02 = No | |  | |  |  |
| **8** | |  |  | | Female=01  Male=02 | 01 =Yes  02 = No | 01 =Yes  02 = No | |  | |  |  |
| **9** | |  |  | | Female=01  Male=02 | 01 =Yes  02 = No | 01 =Yes  02 = No | |  | |  |  |
| **10** | |  |  | | Female=01  Male=02 | 01 =Yes  02 = No | 01 =Yes  02 = No | |  | |  |  |
| *Codes for Q4 Relationship to household head  01=self, 02=spouse, 03=son; 04=Daughter in-law, 05= Daughter in-law; 06=grand son/daughter, 07= brother/Sister; 08= Sister-in-law; 09= Nephew/Niece; 10=father/mother, 01=father/mother in-law, 77= Others (Please specify) | | | | | | | | | | | | |
| **Codes for Q6 marital status  1=never married, 2=married, 3=divorced, 4=separated, 5=widow/widower,6=unmarried | | | | | | | | | | | | |
| ***Codes for Q8 of education  0=Illiterate, 1=Class one; 5=Class five; 10=SSC; 12=HSC; 14=Diploma; 16=graduation; 18= Post-graduate ; 77= Other (Please specify) | | | | | | | | | | | | |
| Agriculture,=1, Aquaculture=2, Both Agril & Aqua=3, , Housewife=04, Business=05, Service (Govt & NGO)=06, skilled labour (mason/carpenter)=07, labour=08, Domestic help=09, Rickshaw/Van driver=10, Daily labour=11; Retired/Old person=12, Unemployed=13, Student=14; Kids=15; Involved in SFVC=16; Others (specify) =77 | | | | | | | | | | | | |
| e.no | Question | | | Codding categories | | | | Code | | Skip | | |
|  | Have you taken consent in the form? | | | 1 =Yes  2 = No | | | |  | |  | | |
|  | Number of HH member? | | | HH members number | | | |  | |  | | |
|  | Type of family | | | 1=Nuclear, 2=Combined | | | |  | |  | | |
|  | Is this HH involed in nutrition intervention programme? | | | 1 =Yes, 2 = No | | | |  | |  | | |
|  | You or your family are belong to which religion? (only one answer) | | | 01=Muslim  02=Hindu  03=Christian  04=Buddist  77=other (Specify)-------- | | | |  | |  | | |
|  | Is there any member of your HH involve in fish production, marketing or any activities of seafood value chain (SFVC)? | | | 1 =Yes  2 = No | | | |  | | If No skip to Ques. No. 8 | | |
|  | If yes are they involve for round the year? | | | 1 =Yes  2 = No | | | |  | |  | | |
|  | If No then where the outmigration happened and for how long? | | | Time : Month | | | |  | |  | | |
|  | What is the main source of drinking water for your household?  (only one answer is expected) | | | 01=Tube-well (personal)  02=Tube-well (Others)  03=Tube-well (shared)  04=Tube-well (govt/community)  05=Tape water  06=Reservoir  07=Pond  08=River/Canal  09=Rain water  10= pond water drink after filtration  11=mineral water  77=Others(Specify) | | | |  | |  | | |
|  | Distance of water source from HH | | | ______________________ yard | | | |  | |  | | |
|  | What is the main source of water for bathing in your household? | | | 01=Tube-well (personal)  02=Tube-well (Others)  03=Tube-well (shared)  04=Tube-well (govt/community)  05=Tape water  06=Reservoir  07=Pond  08=River/Canal  09=Rain water  10= pond water drink after filtration | | | |  | |  | | |
|  | What is the main source of water for cleaning utensils in your household? | | | 01=Tube-well (personal)  02=Tube-well (Others)  03=Tube-well (shared)  04=Tube-well (govt/community)  05=Tape water  06=Reservoir  07=Pond  08=River/Canal  09=Rain water  10= pond water drink after filtration | | | |  | |  | | |
|  | Researcher will look the colour of the tube well | | | 01=Red (Arsenic affected)  02=Green (No arsenic)  03=Not coloured (Not tested)  04=Not applicable | | | |  | |  | | |
|  | What kind of toilet facility does your household have? | | | 01=Modern latrine (water seal)  02=Slab latrine (without water seal)  03=Pit Larine  04=Open latrin | | | |  | |  | | |
|  | Do you share this facilities with other HH? | | | 1 =Yes  2 = No | | | |  | | If No Skip to Ques 16 | | |
|  | If yes with how many families? | | |  | | | |  | |  | | |
|  | Which fuel do you use for cooking? | | | 01=Electricity  02=LPG  03=Natural gas  04=Bio-gas  05=kerosin  06=Charcole  07=wood  08=Cowdung  77=Other (Please specify) | | | |  | |  | | |
|  | What is the main element of floor?  [observe and note] | | | 01=Concrete  02=brick/cement  03=Wood  04=fine mud  05=tali  06=tiels  77=Other (Please specify) | | | |  | |  | | |
|  | What is the main element of wall?  [observe and note] | | | 01=Concrete  02=brick/cement  03=Wood  04=fine mud  05=tali  06=Bamboo  77=Other (Please specify) | | | |  | |  | | |
|  | What is the main element of roof?  [observe and note] | | | 01=Concrete  02=brick/cement  03=Wood  04=fine mud  05=Tin/CI sheet  06=Partex board  07=Bamboo/Grass/Straw  77=Other (Please specify) | | | |  | |  | | |
|  | Do you or your family have the following item/goods? | | | | | | | | | | | |

|  | a. Electricity | | 01 =Yes , 02 = No | |  | | |
| --- | --- | --- | --- | --- | --- | --- | --- |
|  | b. Radio | | 01 =Yes , 02 = No | |  | | |
|  | c. Television | | 01 =Yes , 02 = No | |  | | |
|  | d. Mobile | | 01 =Yes , 02 = No | |  | | |
|  | e. Landphone | | 01 =Yes , 02 = No | |  | | |
|  | f. Freeze | | 01 =Yes , 02 = No | |  | | |
|  | g. Almerah/Wardrobe | | 01 =Yes , 02 = No | |  | | |
|  | h. Table | | 01 =Yes , 02 = No | |  | | |
|  | i. Chair | | 01 =Yes , 02 = No | |  | | |
|  | j. Watch | | 01 =Yes , 02 = No | |  | | |
|  | k. Bicycle | | 01 =Yes , 02 = No | |  | | |
|  | l. Motor cycle/scooter/tempo | | 01 =Yes , 02 = No | |  | | |
|  | m. Cart | | 01 =Yes , 02 = No | |  | | |
|  | n. Car/truck | | 01 =Yes , 02 = No | |  | | |
|  | o. Boat | | 01 =Yes , 02 = No | |  | | |
|  | p. Rikshaw/Van | | 01 =Yes , 02 = No | |  | | |
|  | **Part-B Household Farming activities**  Questions will be asked mainly to the HH head or the person leading the farming activities at HH level  Land Information: | | | | | | |
| Type | Homestead with garden (a) | | Cultivable (b) | Business/ enterprise (c) | | Other (specify) (d) | Total (a+b+c+d) |
|  | Area (decimal) | |  |  | |  |  |
|  | **Ownership of farming land** | | | | | | |
| Ownership category | | Number of land unit | Area (decimal) | | | | |
| Own (A) | |  | decimal | | | | |
| Leased-in (B) | |  | decimal | | | | |
| Total (A+B) | |  | decimal | | | | |
| Leased-out* | |  | decimal | | | | |
| * *Leased-out area will not added to total land areas as this is not under the custody of HH. | | | | | | | |

| 1. **Utility of cultivable land**t | | | | | | | | | | | | | | |
| --- | --- | --- | --- | --- | --- | --- | --- | --- | --- | --- | --- | --- | --- | --- |
| **Purpose** | | | **Only** | |  | | **Concurrent/alternate** | | | | |  | | |
| Rice | | | 01 =Yes , 02 = No | |  | | 01=only, 02=concurrent  03=alternate, 04=not applicable | | | | |  | | |
| Fish | | | 01 =Yes , 02 = No | |  | | 01=only, 02=concurrent  03=alternate, 04=not applicable | | | | |  | | |
| Rice+Fish | | | 01 =Yes , 02 = No | |  | | 01=only, 02=concurrent  03=alternate, 04=not applicable | | | | |  | | |
| Rice+Fish+Dyke crop | | | 01 =Yes , 02 = No | |  | | 01=only, 02=concurrent  03=alternate, 04=not applicable | | | | |  | | |
| Fish+Dyke crop | | | 01 =Yes , 02 = No | |  | | 01=only, 02=concurrent  03=alternate, 04=not applicable | | | | |  | | |
| Rice+Dyke crop | | | 01 =Yes , 02 = No | |  | | 01=only, 02=concurrent  03=alternate, 04=not applicable | | | | |  | | |
| 1. **Fish Production Area** | | | | | | | | | | | | | |  |
| Fish production unit | Location/distance from HH | | | No of unit | Area (deci) | Ownership | | | If owned | | Users HH number* | | Utility |  |
| Pond | Yard/__/__/ | | |  | /__/__/ | Own=01  Leased-in=02  Both=03 | | | Single=01  Multiple=02  Both=03 | |  | | Fish=01  HH use=02  Both=03 |  |
| *Gher* | Yard /__/__/ | | |  | /__/__/ | Own=01  Leased-in=02  Both=03 | | | Single=01  Multiple=02  Both=03 | |  | | Fish=01  Crop=02  Both=03 |  |
| 1. Water salinity* of *gher*: According to the farmer perception: | | | | | | | | | | | | | |  |
| Q# | | Salinity level | | | | | | PPT level (perception) | | PPT level (Actual measurement) | | | |  |
| Water salinity level perception by the farmer | | High=01  Medium=02  Low=03  Freshwater=4 | | | | | |  | |  | | | |  |
| [this perception will be then matched with actual salinity data collected over the year] | | | | | | | | | | | | | |  |
|  | | Average water depth in *gher* | | | | | | cm /__/__/ | | If not code 00 | | | |  |
|  | | Average water depth in *pond* | | | | | | cm /__/__/ | | If not code 00 | | | |  |

| 1. Main water Source in gher | | Sources | | | Remarks | |
| --- | --- | --- | --- | --- | --- | --- |
| Water source in gher | | River=1  Canal=2  Rain=3  Pond=4  Other gher=5  Deep tubewell =06  Shallow tube-well=07  Others=77 (Please specify) | | |  | |
|  | Year of experiences of fish farming? __________________ Years | | | | | |
|  | **Fish Production:** Amount of fish produced last year? | | | | | |
| Unit | Amount sold in market (a) | | Amount consumed at HH (b) | Amount Gifted to others (c) | | Amount (kg) (a+b+c) |
| 1. Gher |  | |  |  | |  |
| 1. pond |  | |  |  | |  |

|  | | Was these amount average/high/below range of production? : Average=1, Lower=02, Higher=03, 04= not applicable | | | | | | | |
| --- | --- | --- | --- | --- | --- | --- | --- | --- | --- |
|  | | **Rice and dyke vegetable production and destination**t | | | | | | | |
| Species/Variety | | Destination | | | | Total amount (kg) | Production cost (BDT) e | Return (Net Return) (f) If not code 00 | |
|  |  | Market (a) | Family (b) | | Gift (c) | d(a+b+c) |  |  |  |
| Rice | |  |  | |  |  |  |  | |
| Vegetables | |  |  | |  |  |  |  | |
| Total | |  |  | |  |  |  |  | |
|  | Do you have any loan | | | 01=Yes  02=No if not skip next question | | | | |  |

|  | If yes then soures   1. Bank 2. NGO 3. Agent 4. Wholesaler 5. Co-operative 6. Others | 01=Yes 02=No  01=Yes 02=No  01=Yes 02=No  01=Yes 02=No  01=Yes 02=No  01=Yes 02=No |  |
| --- | --- | --- | --- |
| 37. | Main reason of loan | A.___________  B.___________  C.___________  D____________ |  |
| 38. | Amount of money   1. Bank 2. NGO 3. Agent 4. Wholesaler 5. Co-operative 6. Others | In thousands |  |
| 39. | Way of loan back   1. Bank 2. NGO 3. Agent 4. Wholesaler 5. Co-operative 6. Others | Total=01 premium=02  Total=01 premium=02  Total=01 premium=02  Total=01 premium=02  Total=01 premium=02  Total=01 premium=02 |  |
| 40. | Interest rate   1. Bank 2. NGO 3. Agent 4. Wholesaler 5. Co-operative 6. Others | %  %  %  %  %  _% |  |

**Food consumption pattern of adolescent girls at HH level:**

| Se.no | Question | Codding categories | Code | Skip |
| --- | --- | --- | --- | --- |
|  | How are you today? | 01 = Fine , 02 = Not good  03 = Sick |  | If code 01 skip to 52 |
|  | If you are unwell for how many days you are sick? | days |  |  |
|  | Does this illness hamper your food consumption? | 01 = Yes  02 = No |  |  |
|  | Have you suffered from any diseases in the last 15 days? | 01 = Yes  02 = No |  |  |
|  | If yes what type of problem/disease? | 01 = Fever/caugh  02 = diarrhoea  03 = Typhoid  04 = Skin disease  77 = Other (Please specify) | Yes=1 No=2  Yes=1 No=2  Yes=1 No=2  Yes=1 No=2  Yes=1 No=2 |  |
| **Ask the girl question about the nutritional knowledge level** | | | | |
| 12. | Do you know how to prevent diarhoea? | 01 = Yes  02 = No |  |  |
| 13. | If yes then how? | 1. _________________ 2. _________________ 3. _________________ | | |
| 14. | Do you know what is annemea? | 01 = Yes  02 = No |  |  |
| 15. | If the answer is yes then why it happened? | 1. _________________ 2. _________________ 3. _________________ | | |
| 16. | Which fish contains Vitamin-A? | 1. _________________ 2. _________________ 3. _________________ | | |
| 17. | What type (size) of fish contain higher nutrition? | 01=large fish  02= Small fish |  |  |
| 18. | Which source of fish contain nutrition? (more than one answer) | 01=Marine fish  02=Freshwater fish  03=Shell fish  04=Fish byproducts  098=Unknown |  |  |

| 51. | Food consumption of Adolescent girls:  Before working on this issue the research will bring the list of the fish that was addressed at HH level and it will help the researcher to assist/remind the interviewee about fish consumption in the last 7 days. Here the fish size will also standardized and photo album will be used as described earlier. The fish species size/portion size in case of small fish will be considered. The fish consumed with head and without head AND eating with soft bone or not will also be consider  **Semi-quantitative food consumption for the last 24 hours:** | | | | | | |
| --- | --- | --- | --- | --- | --- | --- | --- |
|  | Foods | Food taken or not (1) | Name of the serving utensil (2) | Morning  No of serving size  (3) | | Lunch  No of serving size  (4) | Dinner  No of serving size (5) |
| a. | cereals (boiled rice, puffed rice) | 1= Yes  2= No | 1= Bowl  2=Spoon  3=Piece  4=Cup | 1= Big  2=Medium  3=Small  4=didn’t eat |  | 1= Big  2=Medium  3=Small  4=didn’t eat | 1= Big  2=Medium  3=Small  4=didn’t eat |
| b. | Leafy vegetables | 1= Yes  2= No | 1= Bowl  2=Spoon  3=Piece  4=Cup | 1= Big  2=Medium  3=Small  4=didn’t eat | | 1= Big  2=Medium  3=Small  4=didn’t eat | 1= Big  2=Medium  3=Small  4=didn’t eat |
| c. | Non-leafy vegetables | 1= Yes  2= No | 1= Bowl  2=Spoon  3=Piece  4=Cup | 1= Big  2=Medium  3=Small  4=didn’t eat | | 1= Big  2=Medium  3=Small  4=didn’t eat | 1= Big  2=Medium  3=Small  4=didn’t eat |
| d. | pulses (pulses and legumes) | 1= Yes  2= No | 1= Bowl  2=Spoon  3=Piece  4=Cup | 1= Big  2=Medium  3=Small  4=didn’t eat | | 1= Big  2=Medium  3=Small  4=didn’t eat | 1= Big  2=Medium  3=Small  4=didn’t eat |
| e. | meat (chicken and meat products) | 1= Yes  2= No | 1= Bowl  2=Spoon  3=Piece  4=Cup | 1= Big  2=Medium  3=Small  4= didn’t eat | | 1= Big  2=Medium  3=Small  4=didn’t eat | 1= Big  2=Medium  3=Small  4=didn’t eat |
| f. | Egg | 1= Yes  2= No | 1= full  2= half  3=didn’t eat | 1= full  2= half  3=didn’t eat | | 1= full  2= half  3=didn’t eat | 1= full  2= half  3=didn’t eat |
| g. | milk (dairy products) | 1= Yes  2= No | 1= Big  2=Medium  3=Small | 1= Big  2=Medium  3=Small  4=didn’t eat | | 1= Big  2=Medium  3=Small  4=didn’t eat | 1= Big  2=Medium  3=Small  4=didn’t eat |
| h. | beverage (tea, coffee) | 1= Yes  2= No | 1= Big  2=Medium  3=Small | 1= Big  2=Medium  3=Small | | 1= Big  2=Medium  3=Small | 1= Big  2=Medium  3=Small |
| i. | Fruits | 1= Yes  2= No | 1= full  2= half  3=didn’t eat | 1= Big  2=Medium  3=Small  4=didn’t eat | | 1= Big  2=Medium  3=Small  4=didn’t eat | 1= Big  2=Medium  3=Small  4=didn’t eat |
| j. | others (ice-cream, chocolate) | 1= Yes  2= No | 1= Bowl  2=Spoon  3=Piece  4=Cup  5=number | 1= full  2=half  3=didn’t eat | | 1= full  2=half  3=didn’t eat | 1= full  2=half  3=didn’t eat |

* Cooking oil will be assessed by total amount used at HH/no of HH members

| 1. **Semi-quantitative fish consumption for last 7 days** |
| --- |

| **Days** |  | **Fish species** | **Source (Own=1, market=2, Gift=3)** | **Portion =01 Piece =02**  **Full=03** | **Number of piece/portion consumed** | | | **No of head** | **No of tail** | **No of portion consumed with soft bone** | **No of portion consumed without soft bone** |
| --- | --- | --- | --- | --- | --- | --- | --- | --- | --- | --- | --- |
|  |  |  |  |  | **Big** | **Medium** | **Small** |  |  |  |  |
| **D-7 (last day)** | Yes=01  No=02 |  |  |  |  |  |  |  |  |  |  |
|  |  |  |  |  |  |  |  |  |  |  |  |
|  |  |  |  |  |  |  |  |  |  |  |  |
| **D-6** | Yes=01  No=02 |  |  |  |  |  |  |  |  |  |  |
|  |  |  |  |  |  |  |  |  |  |  |  |
|  |  |  |  |  |  |  |  |  |  |  |  |
| **D-5** | Yes=01  No=02 |  |  |  |  |  |  |  |  |  |  |
|  |  |  |  |  |  |  |  |  |  |  |  |
|  |  |  |  |  |  |  |  |  |  |  |  |
| **D-4** | Yes=01  No=02 |  |  |  |  |  |  |  |  |  |  |
|  |  |  |  |  |  |  |  |  |  |  |  |
|  |  |  |  |  |  |  |  |  |  |  |  |
| **D-3** | Yes=01  No=02 |  |  |  |  |  |  |  |  |  |  |
|  |  |  |  |  |  |  |  |  |  |  |  |
|  |  |  |  |  |  |  |  |  |  |  |  |
| **D-2** | Yes=01  No=02 |  |  |  |  |  |  |  |  |  |  |
|  |  |  |  |  |  |  |  |  |  |  |  |
|  |  |  |  |  |  |  |  |  |  |  |  |
| **D-1** | Yes=01  No=02 |  |  |  |  |  |  |  |  |  |  |
|  |  |  |  |  |  |  |  |  |  |  |  |
|  |  |  |  |  |  |  |  |  |  |  |  |
