# Supplementary material for: Association of Fish Consumption with the Omega-3 Index and Vitamin D Status among Adolescent Females in the Southwest Coastal Zone of Bangladesh
Source: J Nutr. 2026 May 6;156(6):101520. doi: 10.1016/j.tjnut.2026.101520 (PMC13279382; doi:10.1016/j.tjnut.2026.101520)

**Supplementary Table 1:** Association between weekly fish consumption and O3I among adolescent females across seasons (full model).

| **Characteristics** | **O3I** | | | | **O3I≥4%** | | | |
| --- | --- | --- | --- | --- | --- | --- | --- | --- |
|  | **Model I** | | **Model II** | | **Model III** | | **Model IV** | |
|  | **Coef. [95%CI]** | ***p-value*** | **Coef. [95%CI]** | ***p-value*** | **AOR [95%CI]** | ***p-value*** | **AOR [95%CI]** | ***p-value*** |
| **Dry season (n=295)** |  |  |  |  |  |  |  |  |
| Total fish intake |  |  |  |  |  |  |  |  |
| T1 (≤347 g) | Ref. |  |  |  | Ref. |  |  |  |
| T2 (347-615 g) | 0.27 [-0.01, 0.55] | 0.056 |  |  | 1.61 [0.86, 3.03] | 0.139 |  |  |
| T3 (≥616 g) | 0.57 [0.30, 0.84] | <0.001 |  |  | 2.12 [1.09, 4.11] | 0.027 |  |  |
| Tilapia intake |  |  |  |  |  |  |  |  |
| T1 (≤180 g) |  |  | Ref. |  |  |  | Ref. |  |
| T2 (181-357 g) |  |  | 0.68 [0.42, 0.95] | <0.001 |  |  | 3.12 [1.57, 6.18] | 0.001 |
| T3 (≥358 g) |  |  | 1.08 [0.81, 1.35] | <0.001 |  |  | 3.65 [1.82, 7.32] | <0.001 |
| Crustacean fish intake |  |  |  |  |  |  |  |  |
| T1 (≤9 g) |  |  | Ref. |  |  |  | Ref. |  |
| T2 (10-40 g) |  |  | -0.08 [-0.44, 0.28] | 0.657 |  |  | 1.15 [0.46, 2.89] | 0.765 |
| T3 (≥41 g) |  |  | -0.19 [-0.44, 0.06] | 0.132 |  |  | 1.00 [0.51, 1.93] | 0.989 |
| Small fish intake |  |  |  |  |  |  |  |  |
| T1 (≤3 g) |  |  | Ref. |  |  |  | Ref. |  |
| T2 (4-60 g) |  |  | 0.36 [0.09, 0.63] | 0.009 |  |  | 1.81 [0.86, 3.80] | 0.119 |
| T3 (≥61 g) |  |  | 0.18 [-0.09, 0.44] | 0.188 |  |  | 1.01 [0.51, 2.00] | 0.982 |
| Large fish intake |  |  |  |  |  |  |  |  |
| T1 (≤19 g) |  |  | Ref. |  |  |  | Ref. |  |
| T2 (20-120 g) |  |  | 0.002 [-0.26, 0.27] | 0.986 |  |  | 0.92 [0.45, 1.88] | 0.817 |
| T3 (≥121 g) |  |  | -0.06 [-0.33, 0.21] | 0.655 |  |  | 1.21 [0.58, 2.50] | 0.611 |
| R-squared (%) | 29.60 |  | 40.80 |  | 16.50 |  | 20.40 |  |
| **Wet season (n=260)** |  |  |  |  |  |  |  |  |
| Total fish intake |  |  |  |  |  |  |  |  |
| T1 (≤300 g) | Ref. |  |  |  | Ref. |  |  |  |
| T2 (301-590 g) | 0.86 [0.54, 1.17] | <0.001 |  |  | 2.67 [1.18, 6.07] | 0.019 |  |  |
| T3 (≥590 g) | 1.26 [0.94, 1.59] | <0.001 |  |  | 7.98 [2.69, 23.66] | <0.001 |  |  |
| Tilapia intake |  |  |  |  |  |  |  |  |
| T1 (≤135 g) |  |  | Ref. |  |  |  | Ref. |  |
| T2 (136-378 g) |  |  | 1.14 [0.83, 1.45] | <0.001 |  |  | 7.53 [2.82, 20.13] | <0.001 |
| T3 (≥379 g) |  |  | 1.63 [1.31, 1.94] | <0.001 |  |  | 23.85 [6.25, 90.95] | <0.001 |
| Crustacean fish intake |  |  |  |  |  |  |  |  |
| T1 (≤9 g) |  |  | Ref. |  |  |  | Ref. |  |
| T2 (10-60 g) |  |  | -0.17 [-0.48, 0.14] | 0.287 |  |  | 1.65 [0.51, 5.28] | 0.401 |
| T3 (≥61 g) |  |  | 0.11 [-0.19, 0.40] | 0.475 |  |  | 1.26 [0.48, 3.28] | 0.638 |
| Small fish intake |  |  |  |  |  |  |  |  |
| T1 (≤4 g) |  |  | Ref. |  |  |  | Ref. |  |
| T2 (5-20 g) |  |  | -0.22 [-0.67, 0.23] | 0.34 |  |  | 0.54 [0.14, 2.12] | 0.374 |
| T3 (≥21 g) |  |  | 0.06 [-0.22, 0.34] | 0.667 |  |  | 0.91 [0.33, 2.55] | 0.864 |
| Large fish intake |  |  |  |  |  |  |  |  |
| T1 (≤9 g) |  |  | Ref. |  |  |  | Ref. |  |
| T2 (10-90 g) |  |  | 0.20 [-0.11, 0.52] | 0.205 |  |  | 2.42 [0.8, 7.26] | 0.116 |
| T3 (≥91 g) |  |  | 0.19 [-0.10, 0.48] | 0.191 |  |  | 1.85 [0.71, 4.86] | 0.211 |
| R-squared (%) | 49.30 |  | 56.50 |  | 31.70 |  | 40.26 |  |

AOR: Adjusted odds ratio; CI: Confidence interval; Coef: Coefficient; O3I: Omega-3 Index; Ref: Reference; T: Tertiles of fish consumption.

Models I–II: linear regression (continuous O3I); Models III–IV: logistic regression (O3I ≥ 4%). All models also adjusted for age, religion, dietary diversity, salinity zone, and wealth index. R² indicates variance explained.

**Supplementary Table 2:** Association between weekly fish consumption and vitamin D status among adolescent females across seasons (full model).

| **Characteristics** | **Serum 25(OH)D level (nmol/L)** | | | | **25(OH)D insufficiency (<50nmol/L)** | | | |
| --- | --- | --- | --- | --- | --- | --- | --- | --- |
|  | **Model I** | | **Model II** | | **Model III** | | **Model IV** | |
|  | **Coef. [95%CI]** | ***p-value*** | **Coef. [95%CI]** | ***p-value*** | **AOR [95%CI]** | ***p-value*** | **AOR [95%CI]** | ***p-value*** |
| **Dry season (n=295)** |  |  |  |  |  |  |  |  |
| Total fish intake |  |  |  |  |  |  |  |  |
| T1 (≤347 g) | Ref. |  |  |  | Ref. |  |  |  |
| T2 (347-615 g) | -3.74 [-7.81, 0.33] | 0.072 |  |  | 1.22 [0.63, 2.37] | 0.561 |  |  |
| T3 (≥616 g) | -0.21 [-4.77, 4.35] | 0.928 |  |  | 0.79 [0.39, 1.63] | 0.527 |  |  |
| Tilapia intake |  |  |  |  |  |  |  |  |
| T1 (≤180 g) |  |  | Ref. |  |  |  | Ref. |  |
| T2 (181-357 g) |  |  | 6.92 [2.78, 11.06] | 0.001 |  |  | 0.48 [0.23, 1.03] | 0.058 |
| T3 (≥358 g) |  |  | 0.93 [-3.7, 5.57] | 0.692 |  |  | 1.05 [0.52, 2.11] | 0.890 |
| Crustacean fish intake |  |  |  |  |  |  |  |  |
| T1 (≤9 g) |  |  | Ref. |  |  |  | Ref. |  |
| T2 (10-40 g) |  |  | -2.29 [-8.02, 3.43] | 0.431 |  |  | 1.6 [0.63, 4.06] | 0.318 |
| T3 (≥41 g) |  |  | -0.7 [-4.54, 3.13] | 0.718 |  |  | 0.76 [0.37, 1.54] | 0.443 |
| Small fish intake |  |  |  |  |  |  |  |  |
| T1 (≤3 g) |  |  | Ref. |  |  |  | Ref. |  |
| T2 (4-60 g) |  |  | 0.58 [-4.38, 5.54] | 0.818 |  |  | 1.13 [0.54, 2.36] | 0.750 |
| T3 (≥61 g) |  |  | 3.72 [-0.46, 7.9] | 0.081 |  |  | 0.71 [0.34, 1.47] | 0.350 |
| Large fish intake |  |  |  |  |  |  |  |  |
| T1 (≤19 g) |  |  | Ref. |  |  |  | Ref. |  |
| T2 (20-120 g) |  |  | -4.87 [-9.39, -0.35] | 0.035 |  |  | 1.26 [0.6, 2.68] | 0.542 |
| T3 (≥121 g) |  |  | -0.57 [-5.33, 4.19] | 0.814 |  |  | 0.81 [0.37, 1.77] | 0.600 |
| R-squared (%) | 26.40 |  | 30.00 |  | 10.37 |  | 12.86 |  |
| **Wet season (n=260)** |  |  |  |  |  |  |  |  |
| Total fish intake |  |  |  |  |  |  |  |  |
| T1 (≤300 g) | Ref. |  |  |  | Ref. |  |  |  |
| T2 (301-590 g) | 4.38 [-0.05, 8.81] | 0.053 |  |  | 0.68 [0.34, 1.36] | 0.279 |  |  |
| T3 (≥590 g) | 5.06 [0.54, 9.59] | 0.028 |  |  | 0.48 [0.23, 1.00] | 0.050 |  |  |
| Tilapia intake |  |  |  |  |  |  |  |  |
| T1 (≤135 g) |  |  | Ref. |  |  |  | Ref. |  |
| T2 (136-378 g) |  |  | 4.50 [0.29, 8.72] | 0.036 |  |  | 0.66 [0.31, 1.42] | 0.294 |
| T3 (≥379 g) |  |  | 7.96 [3.49, 12.42] | 0.001 |  |  | 0.25 [0.11, 0.57] | 0.001 |
| Crustacean fish intake |  |  |  |  |  |  |  |  |
| T1 (≤9 g) |  |  | Ref. |  |  |  | Ref. |  |
| T2 (10-60 g) |  |  | 4.16 [-0.77, 9.09] | 0.098 |  |  | 0.69 [0.31, 1.54] | 0.363 |
| T3 (≥61 g) |  |  | -0.86 [-5.05, 3.32] | 0.685 |  |  | 0.61 [0.29, 1.30] | 0.201 |
| Small fish intake |  |  |  |  |  |  |  |  |
| T1 (≤4 g) |  |  | Ref. |  |  |  | Ref. |  |
| T2 (5-20 g) |  |  | 4.81 [-2.41, 12.03] | 0.191 |  |  | 0.21 [0.06, 0.78] | 0.020 |
| T3 (≥21 g) |  |  | 1.86 [-2.48, 6.2] | 0.399 |  |  | 0.74 [0.36, 1.52] | 0.417 |
| Large fish intake |  |  |  |  |  |  |  |  |
| T1 (≤9 g) |  |  | Ref. |  |  |  | Ref. |  |
| T2 (10-90 g) |  |  | -0.07 [-4.16, 4.03] | 0.975 |  |  | 1.07 [0.49, 2.37] | 0.859 |
| T3 (≥91 g) |  |  | -0.93 [-4.9, 3.05] | 0.647 |  |  | 1.44 [0.7, 2.96] | 0.324 |
| R-squared (%) | 35.80 |  | 39.70 |  | 17.05 |  | 22.25 |  |

AOR: Adjusted odds ratio; CI: Confidence interval; Coef: Coefficient; Ref: Reference; T: Tertiles of fish consumption; 25(OH)D: 25-hydroxyvitamin D.

Models I–II: linear regression (continuous serum vitamin D level); Models III–IV: logistic regression (vitamin D insufficiency). All models also adjusted for age, religion, dietary diversity, salinity zone, and wealth index. R² indicates variance explained.

**Supplementary Table 3:** Baseline characteristics by data completeness (sensitivity analysis).

| **Variable** | **Complete (n=260)** | **Missing wet season (n=35)** | ***p-value*** |
| --- | --- | --- | --- |
| Age in years [Mean, SD] | 13.83±1.43 | 14.57±1.44 | 0.004 |
| Education level |  |  |  |
| Up to primary | 48 (18.46) | 6 (17.14) | 0.85 |
| Secondary | 212 (81.54) | 29 (82.86) |  |
| Religion |  |  |  |
| Muslim | 147 (56.54) | 28 (80) | 0.008 |
| Hindu | 113 (43.46) | 7 (20) |  |
| Wealth index |  |  |  |
| Poorest | 52 (20.00) | 7 (20.00) | 0.942 |
| Poorer | 52 (20.00) | 7 (20.00) |  |
| Middle | 53 (20.38) | 8 (22.86) |  |
| Richer | 52 (20.00) | 5 (14.29) |  |
| Richest | 51 (19.62) | 8 (22.86) |  |
| Dietary diversity Score | 3.85 (0.89) | 3.74 (0.78) | 0.498 |
| Tilapia intake (g/week) | 260 (145, 425) | 246 (170, 427) | 0.823 |
| Crustacean intake (g/week) | 62.9±121.1 | 75.6±216.8 | 0.464 |
| Large fish intake (g/week) | 60 (0, 140) | 70 (0, 180) | 0.492 |
| Small fish intake (g/week) | 20 (0, 80) | 30 (0, 110) | 0.224 |
| Total fish intake (g/week) | 464.5 (315, 660) | 560 (300, 757) | 0.382 |
| EPA (%) | 0.44 (0.31, 0.62) | 0.50 (0.40, 0.60) | 0.191 |
| DHA (%) | 2.76 (2.24, 3.18) | 2.80 (2.32, 3.10) | 0.861 |
| EPA+DHA in whole blood (%) | 3.25 (2.63, 3.78) | 3.35 (2.80, 3.64) | 0.914 |
| O3I (%) | 4.34 (3.48, 5.08) | 4.49 (3.72, 4.89) | 0.914 |
| 25(OH)D level (nmol/L) | 60.35 (48.8, 70.70) | 62.60 (50.60, 72.30) | 0.319 |
| 25(OH)D insufficiency | 69 (26.54) | 6 (17.14) | 0.231 |

**DHA,** docosahexaenoic acid; **EPA,** eicosapentaenoic acid; **O3I,** Omega-3 index (EPA+ DHA in erythrocytes).

*p-values* were derived using the Wilcoxon rank-sum test or independent sample t test for continuous variables while the chi-square test of independence for categorical variables.

**Supplementary Table 4:** Sensitivity analysis: Association between weekly fish consumption and O3I among adolescent females (12-16 years) during dry season (complete case analysis, n = 260).

| **Characteristics** | **O3I** | | | | **O3I≥4%** | | | |
| --- | --- | --- | --- | --- | --- | --- | --- | --- |
|  | **Model I** | | **Model II** | | **Model III** | | **Model IV** | |
|  | **Coef. [95%CI]** | ***p-value*** | **Coef. [95%CI]** | ***p-value*** | **AOR [95%CI]** | ***p-value*** | **AOR [95%CI]** | ***p-value*** |
| Total fish intake |  |  |  |  |  |  |  |  |
| T1 (≤345 g) | Ref. |  |  |  | Ref. |  |  |  |
| T2 (346-610 g) | 0.15 [-0.15, 0.45] | 0.323 |  |  | 1.42 [0.73, 2.79] | 0.306 |  |  |
| T3 (≥611 g) | 0.57 [0.28, 0.87] | <0.001 |  |  | 1.81 [0.90, 3.65] | 0.096 |  |  |
| Tilapia intake |  |  |  |  |  |  |  |  |
| T1 (≤175 g) |  |  | Ref. |  |  |  | Ref. |  |
| T2 (176-355 g) |  |  | 0.60 [0.31, 0.88] | <0.001 |  |  | 2.83 [1.36, 5.89] | 0.005 |
| T3 (≥356 g) |  |  | 1.10 [0.80, 1.40] | <0.001 |  |  | 3.48 [1.62, 7.47] | 0.001 |
| Crustacean fish intake |  |  |  |  |  |  |  |  |
| T1 (≤9 g) |  |  | Ref. |  |  |  | Ref. |  |
| T2 (10-40 g) |  |  | _ 0.14 [-0.54, 0.27] | 0.502 |  |  | 1.06 [0.41, 2.78] | 0.898 |
| T3 (≥41 g) |  |  | _ 0.14 [-0.40, 0.14] | 0.335 |  |  | 1.22 [0.60, 2.48] | 0.579 |
| Small fish intake |  |  |  |  |  |  |  |  |
| T1 (≤3 g) |  |  | Ref. |  |  |  | Ref. |  |
| T2 (4-55 g) |  |  | 0.46 [0.17, 0.76] | 0.002 |  |  | 2.45 [1.07, 5.62] | 0.034 |
| T3 (≥56 g) |  |  | 0.13 [-0.16, 0.42] | 0.375 |  |  | 0.94 [0.46, 1.92] | 0.872 |
| Large fish intake |  |  |  |  |  |  |  |  |
| T1 (≤19 g) |  |  | Ref. |  |  |  | Ref. |  |
| T2 (20-120 g) |  |  | 0.08 [-0.21, 0.37] | 0.588 |  |  | 0.97 [0.45, 2.09] | 0.93 |
| T3 (≥121 g) |  |  | _0.5 [-0.35, 0.25] | 0.742 |  |  | 1.30 [0.60, 2.80] | 0.502 |
| R-squared | 32% |  | 42% |  | 16.10% |  | 20.50% |  |

AOR: Adjusted odds ratio; CI: Confidence interval; Coef: Coefficient; O3I: Omega-3 Index; Ref: Reference; T: Tertiles of fish consumption.

Models I–II: linear regression (continuous O3I); Models III–IV: logistic regression (O3I ≥ 4%). All models also adjusted for age, religion, dietary diversity, salinity zone, and wealth index. R² indicates variance explained.

**Supplementary Table 5:** Sensitivity analysis: Association between weekly fish consumption and vitamin D status among adolescent females (12-16 years) during dry season (complete case analysis, n = 260).

| **Characteristics** | **Serum 25(OH)D level (nmol/L)** | | | | **25(OH)D insufficiency (<50nmol/L)** | | | |
| --- | --- | --- | --- | --- | --- | --- | --- | --- |
|  | **Model I** | | **Model II** | | **Model III** | | **Model IV** | |
|  | **Coef. [95%CI]** | ***p-value*** | **Coef. [95%CI]** | ***p-value*** | **AOR [95%CI]** | ***p-value*** | **AOR [95%CI]** | ***p-value*** |
| Total fish intake |  |  |  |  |  |  |  |  |
| T1 (≤345 g) | Ref. |  |  |  | Ref. |  |  |  |
| T2 (346-610 g) | -4.1 [-8.54, 0.33] | 0.069 |  |  | 1.25 [0.62, 2.52] | 0.535 |  |  |
| T3 (≥611 g) | -0.77 [-5.62, 4.09] | 0.756 |  |  | 0.93 [0.44, 1.94] | 0.840 |  |  |
| Tilapia intake |  |  |  |  |  |  |  |  |
| T1 (≤175 g) |  |  | Ref. |  |  |  | Ref. |  |
| T2 (176-355 g) |  |  | 5.93 [1.45, 10.40] | 0.01 |  |  | 0.45 [0.21, 0.99] | 0.048 |
| T3 (≥356 g) |  |  | 1.06 [-4.39, 6.52] | 0.701 |  |  | 1.06 [0.50, 2.24] | 0.884 |
| Crustacean fish intake |  |  |  |  |  |  |  |  |
| T1 (≤9 g) |  |  | Ref. |  |  |  | Ref. |  |
| T2 (10-40 g) |  |  | -1.94 [-8.16, 4.28] | 0.539 |  |  | 1.58 [0.61, 4.11] | 0.346 |
| T3 (≥41 g) |  |  | -0.25 [-4.47, 3.98] | 0.909 |  |  | 0.74 [0.35, 1.56] | 0.434 |
| Small fish intake |  |  |  |  |  |  |  |  |
| T1 (≤3 g) |  |  | Ref. |  |  |  | Ref. |  |
| T2 (4-55 g) |  |  | 2.13 [-2.70, 7.33] | 0.365 |  |  | 0.95 [0.42, 2.12] | 0.902 |
| T3 (≥56 g) |  |  | 2.70 [-2.10, 7.50] | 0.268 |  |  | 0.74 [0.35, 1.54] | 0.415 |
| Large fish intake |  |  |  |  |  |  |  |  |
| T1 (≤19 g) |  |  | Ref. |  |  |  | Ref. |  |
| T2 (20-120 g) |  |  | -5.41 [-10.21, -0.60] | 0.028 |  |  | 1.13 [0.51, 2.51] | 0.764 |
| T3 (≥121 g) |  |  | -1.38 [-6.23, 3.47] | 0.576 |  |  | 0.89 [0.40, 1.98] | 0.776 |
| R-squared | 26% |  | 28.81% |  | 9.00% |  | 11.43% |  |

AOR: Adjusted odds ratio; CI: Confidence interval; Coef: Coefficient; Ref: Reference; T: Tertiles of fish consumption; 25(OH)D: 25-hydroxyvitamin D.

Models I–II: linear regression (continuous serum vitamin D level); Models III–IV: logistic regression (vitamin D insufficiency). All models also adjusted for age, religion, dietary diversity, salinity zone, and wealth index. R² indicates variance explained.

**Supplementary Figure 1:** Scatterplot of omega-3 index (%) and total amount of fish consumption (g) in the last seven days during (A) dry season and (B) wet season.


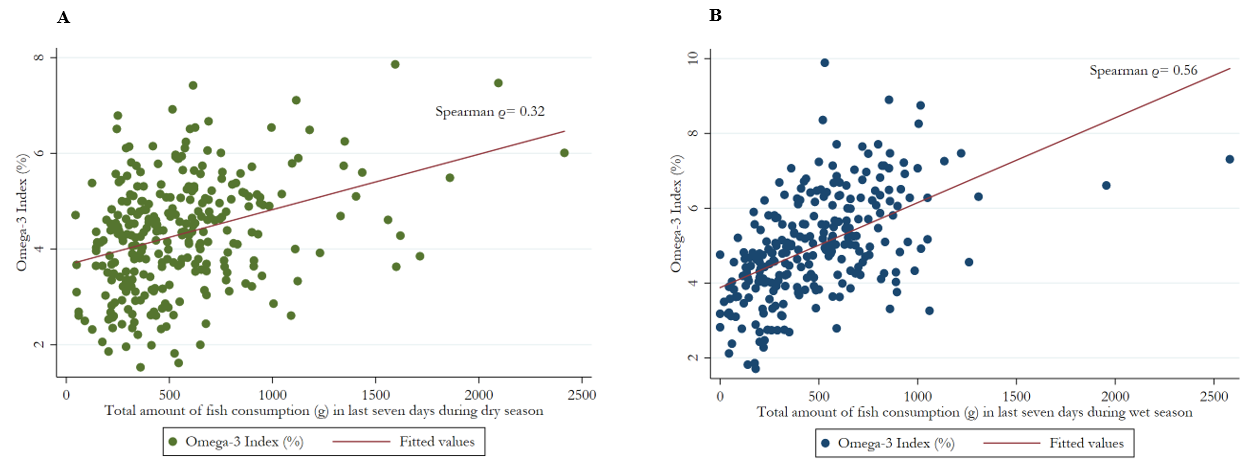


**Supplementary Figure 2:** Scatterplot of omega-3 index (%) and tilapia fish consumption (g) in the last seven days during (A) dry season and (B) wet season.

**(b)**

**
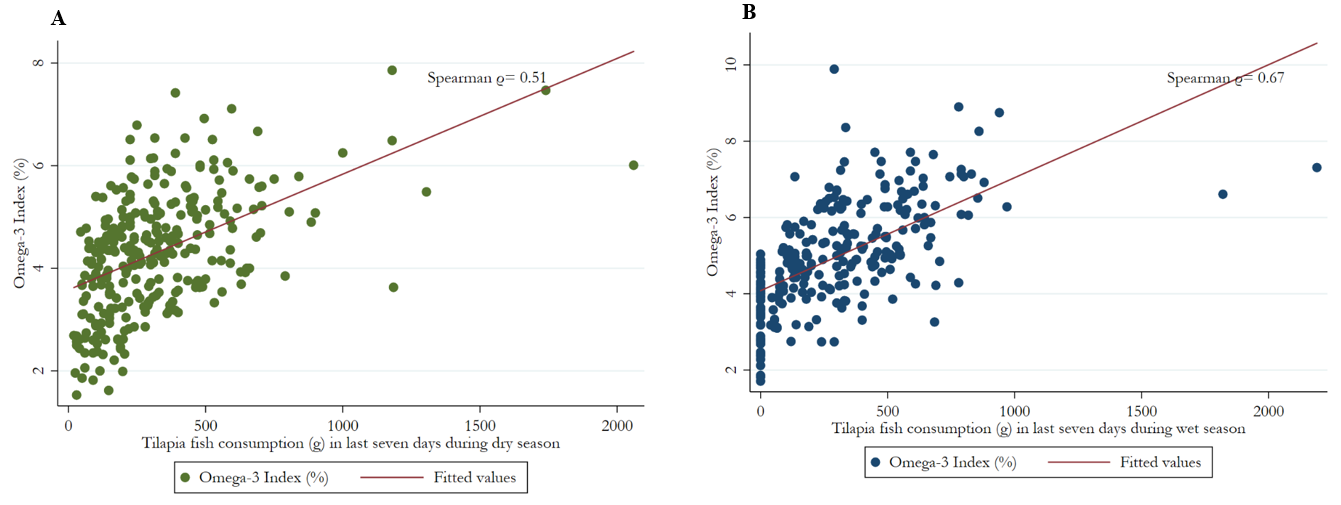
**

**Supplementary Figure 3:** Scatterplot of omega-3 index (%) and crustacean fish consumption (g) in the last seven days during (A) dry season and (B) wet season.


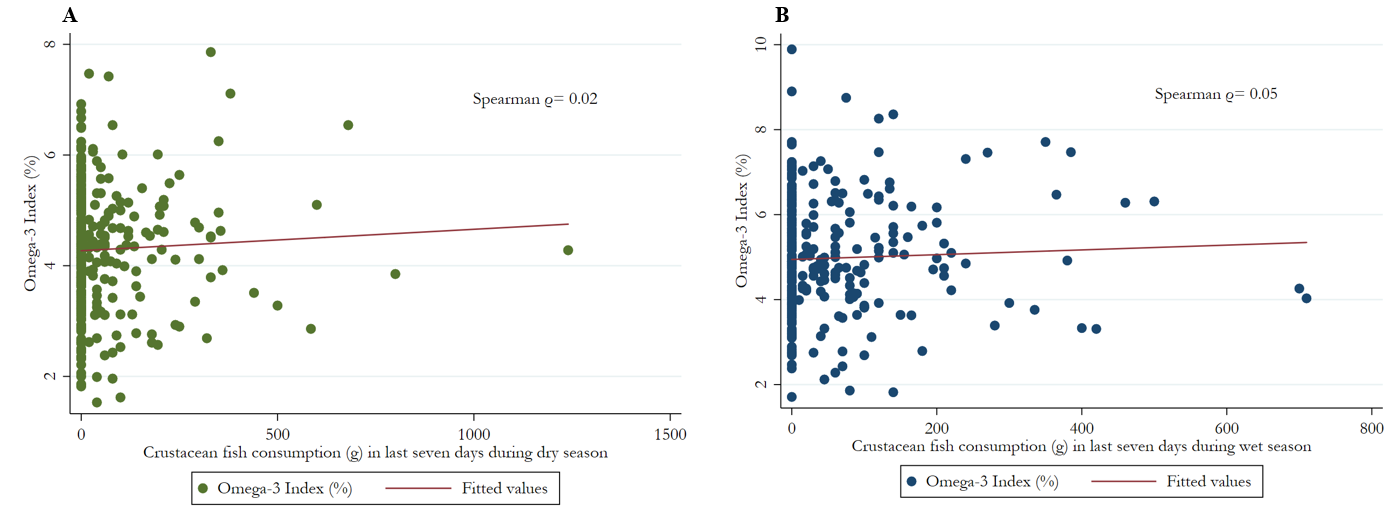


**Supplementary Figure 4:** Scatterplot of omega-3 index (%) and large fish consumption (g) in the last seven days during (A) dry season and (B) wet season.

**
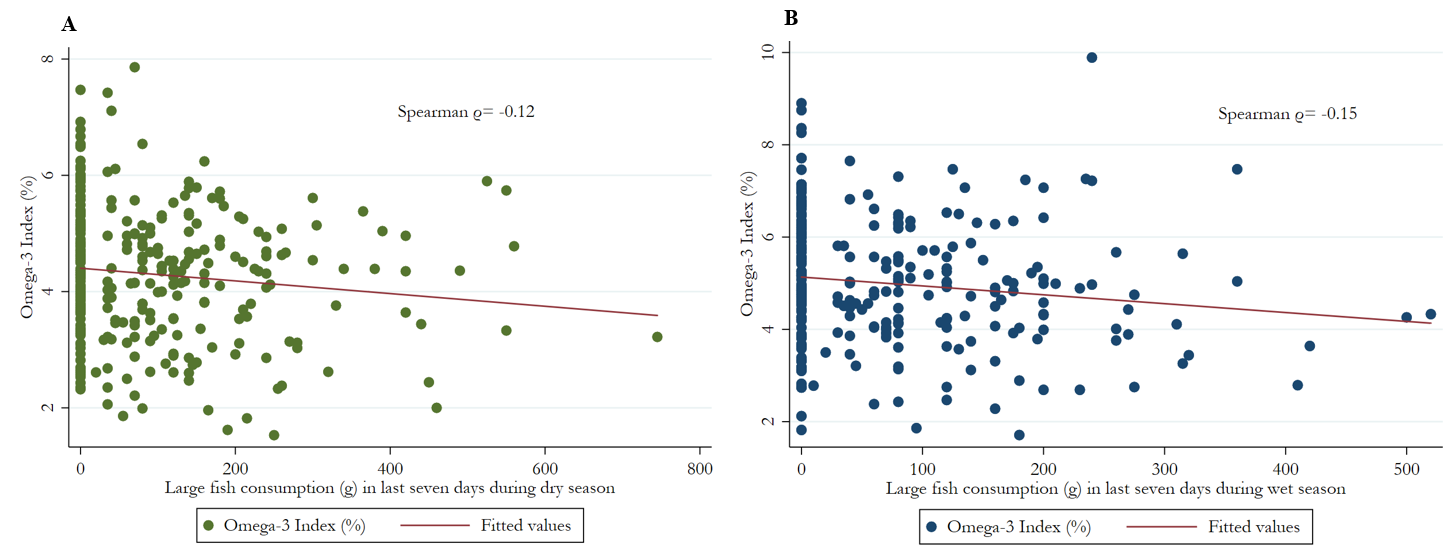
**

**Supplementary Figure 5:** Scatterplot of omega-3 index (%) and small fish consumption (g) in the last seven days during (A) dry season and (B) wet season.


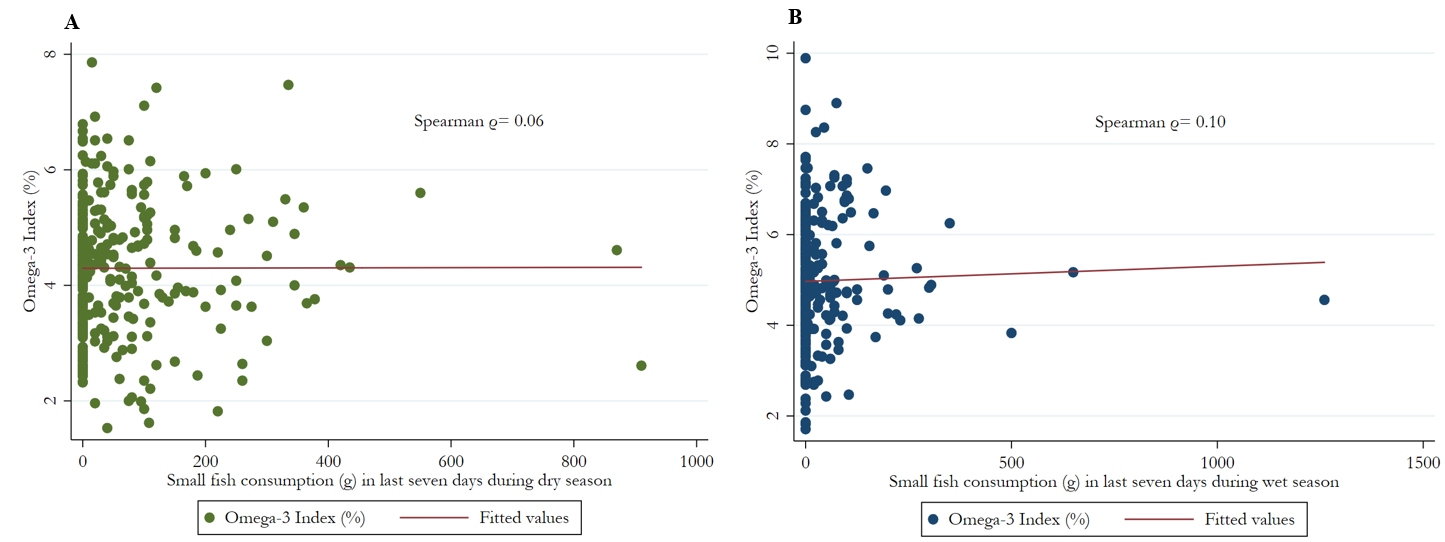

Supplement: Multimedia component 2 [file mmc2.docx]
